# Supplementary material for: Optineurin is an adaptor protein for ubiquitinated substrates in Golgi membrane-associated degradation
Source: Nat Commun. 2025 Oct 20;16:8966. doi: 10.1038/s41467-025-64400-3 (PMC12537940; doi:10.1038/s41467-025-64400-3)
Supplement: Supplementary file 1 — Supplementary Information [file 41467_2025_64400_MOESM1_ESM.pdf]

Supplementary Figure

Yoichi Nibe-Shirakihara[1#], Shinya Honda[1], Satoko Arakawa[1], Satoru Torii[1], Hajime Tajima Sakurai[1], Hirofumi Yamaguchi[1], Shigeru Oshima[2], Ryuichi Okamoto[2], Michal Lazarou[3,4,5], Hideshi Kawakami[6], and Shigeomi Shimizu[1#]

[1] Department of Pathological Cell Biology, Advanced Research Initiative, Institute of Integrated Research, Institute of Science Tokyo, 2-3-10 Kanda Surugadai, Chiyoda-ku, Tokyo 101-0062, Japan

[2] Department of Gastroenterology and Hepatology, Graduate School, Institute of Science Tokyo, 1-5-45 Yushima, Bunkyo-ku, Tokyo 113-8510, Japan

[3] Department of Biochemistry and Molecular Biology, Biomedicine Discovery Institute, Monash University, Melbourne 3800, Australia

[4] Walter and Eliza Hall Institute of Medical Research, Parkville, Victoria, Australia.

[5] Department of Biochemistry and Molecular Biology, Biomedicine Discovery Institute, Monash University, Melbourne, Australia.

[6] Department of Medical Biology, University of Melbourne, Melbourne, Victoria, Australia.

[7]Department of Epidemiology, Research Institute for Radiation Biology and Medicine, Hiroshima University, Hiroshima 734-8553, Japan

Supplementary Figure 1

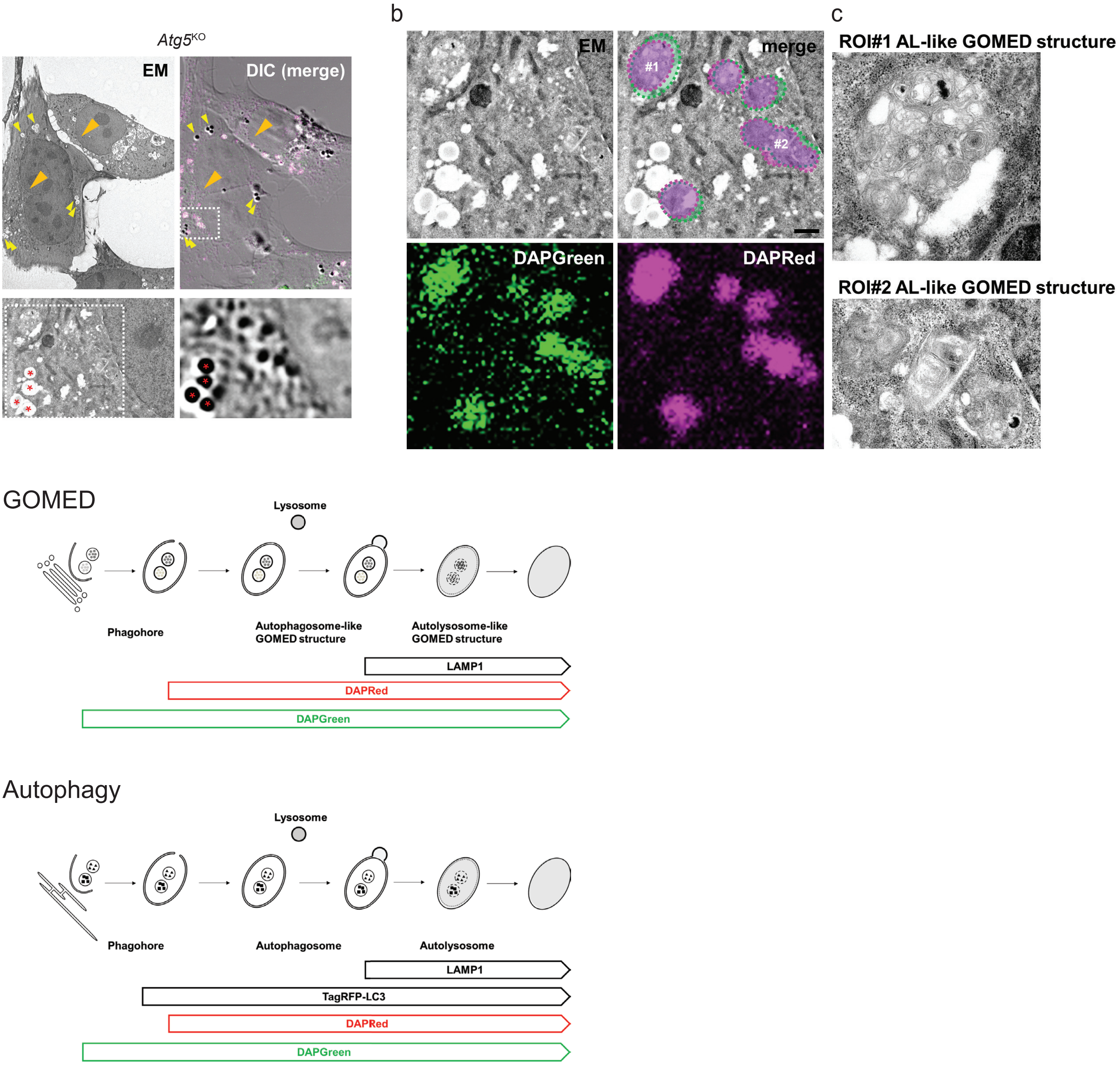

Supplementary Figure 1. Identification of GOMED structures by DAPGreen and DAPRed

a–c. *Atg5*^{KO} MEFs were preincubated with DAPGreen (0.1 μM) and DAPRed (0.1 μM), and treated with etoposide (10 μM) for 10 hr. Then, cells were fixed with 0.75% paraformaldehyde/1.5% glutaraldehyde, and their images were observed by fluorescence microscopy. Cells were subsequently fixed with 1% OsO4 and observed by EM. **a.** DIC and EM images were adjusted for the edge of the plasma membrane, nucleoli (large orange arrowheads), and lipid droplets (small yellow arrowheads). ROIs are indicated by the squares, and magnified images are shown in the lower panels. **b.** Fluorescence signals and EM image of the ROI (lower panel in **a**) are shown. In the upper right image, EM and fluorescence images are merged, and fluorescent signals are traced by dashed circles. Bars = 1 μm. **c.** Magnified EM images of structures #1 and #2 in **b**. CLEM analysis demonstrated that red/green puncta were merged with autolysosome-like GOMED structures. **d.** Schematic diagram of the colocalization of DAPGreen and DAPRed with the various GOMED and autophagic structures. DAPGreen recognizes autophagic and GOMED vacuoles from early phagophores to autolysosomes and autolysosome-like GOMED structures, respectively. DAPRed recognizes autophagic and GOMED vacuoles from late phagophores to autolysosomes and autolysosome-like GOMED structures, respectively. LC3 recognizes autophagic vacuoles, but not GOMED vacuoles. This scheme was designed and created by the authors using Microsoft PowerPoint 365.

Supplementary Figure 2

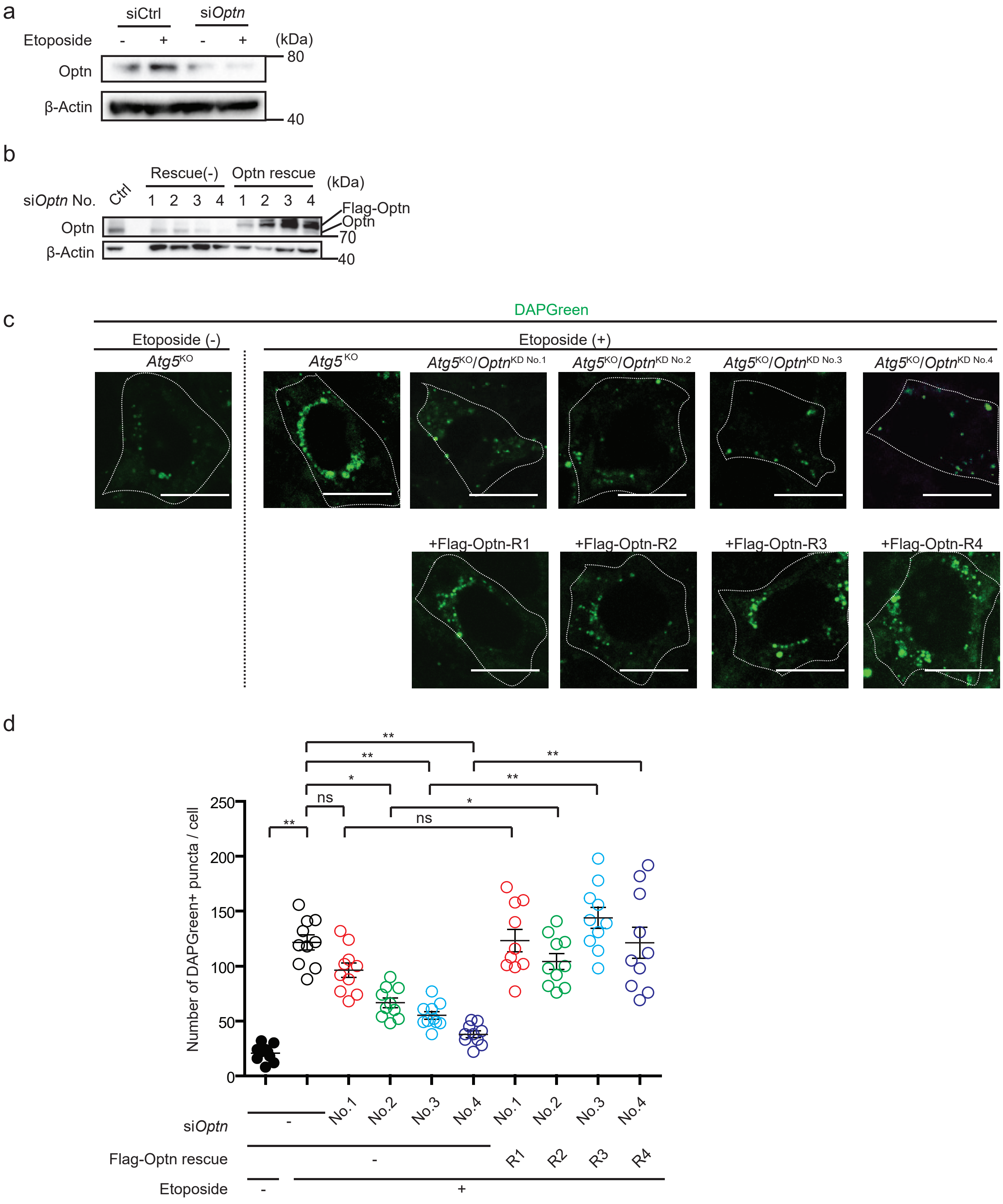

Supplementary Figure 2. OPTN is required for GOMED

a. Successful knockdown of *Optn*. *Optn* was knocked down using siRNAs in *Atg5*^{KO} MEFs. Cells were then treated with etoposide (10 μM) or left untreated for 12 hr. Expression of each protein was analyzed by western blotting. β-actin was used as a loading control. **b–d.** Verification of the effects of the four types of siRNA and their respective resistant gene. *Optn* was knocked down by 4 different types of siRNA in *Atg5*^{KO} MEFs with or without expression of their respective resistant gene after 24 hr. Then, cells were cultured for 24 hr, and the expression of Optn was analyzed by western blotting (**b**). Cells were also treated with etoposide (10 μM) for 12 hr followed by analysis of GOMED using DAPGreen (**c, d**). In (**b**), the number indicates cells transfected with each siOptn (–Rescue) and cells with siOptn plus its respective resistant gene (Optn rescue). In (**c**), representative image of a cell transfected with each siOptn, and a cell with siOptn plus its respective resistant gene. Bar = 10 μm. In (**d**), the number of DAPGreen puncta per cell was calculated. Data are shown as the mean ± SE (n = 10 cells). Comparisons were performed using one-way ANOVA followed by the Tukey's *post-hoc* test. *$p < 0.05$; **$p < 0.01$; ns: not significant Data are representative of two independent experiments in (**a, b and d**).

Supplementary Figure 3

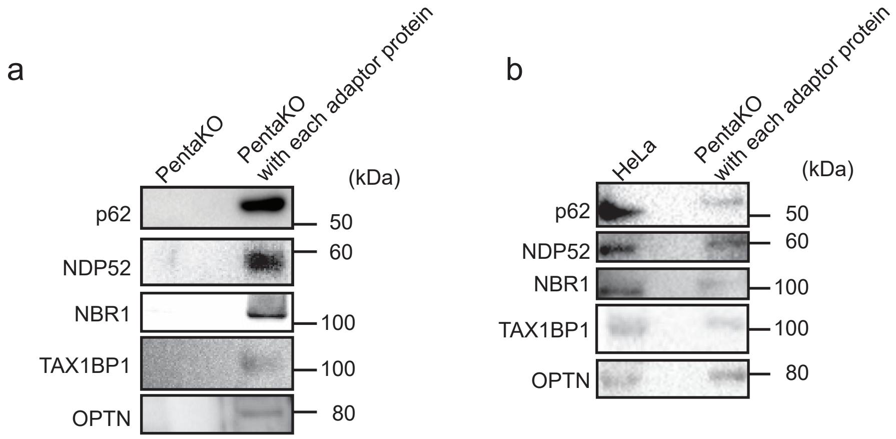

Supplementary Figure 3. Expression of adaptor proteins in PentaKO cells

a. PentaKO cells were transfected with plasmids encoding a Myc (3×)-tagged adaptor protein (p62/SQSTM1, NDP52, NBR1, TAX1BP1, or OPTN). After 24 hr, cells were lysed and immunoblotted using antibodies against the indicated protein. **b.** PentaKO cells were transfected with plasmids encoding a Myc (3×)-tagged adaptor protein (p62/SQSTM1, NDP52, NBR1, TAX1BP1, or OPTN). After 24 hr, HeLa cells and these transfected cells were lysed and immunoblotted using antibodies against the indicated protein. Data are representative of two independent experiments in (**a and b**).

Supplementary Figure 4

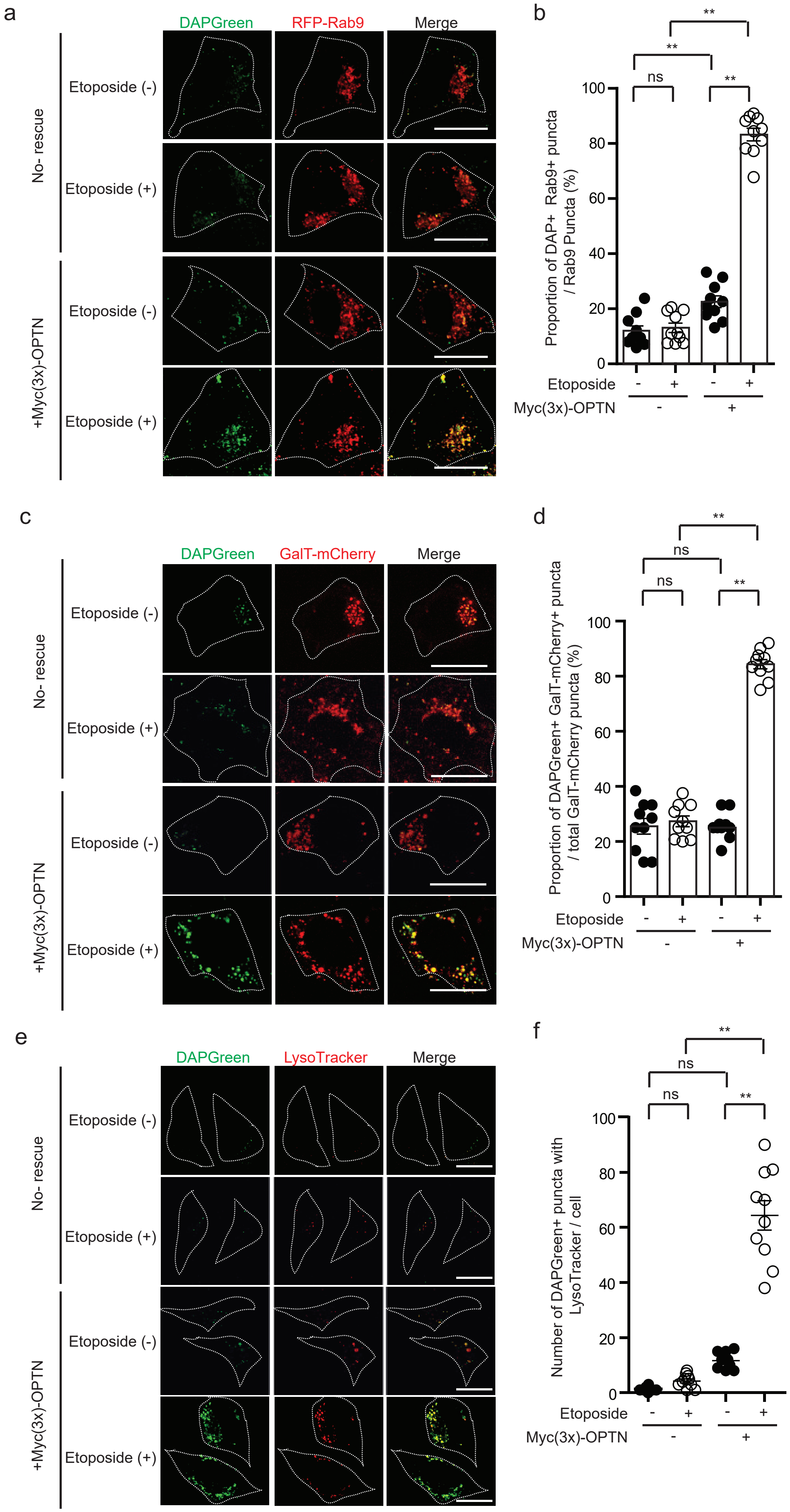

Supplementary Figure 4. Colocalization analysis of DAPGreen puncta with various GOMED markers

a, b. PentaKO cells were transfected with plasmids encoding RFP-Rab9 and Myc (3×)-tagged-OPTN for 24 hr. The cells were treated with etoposide (10 μM) for 12 hr, and then GOMED was analyzed using DAPGreen, and RFP-Rab9 was observed. In (**b**), the proportion of Rab9 puncta that colocalize with DAPGreen was analyzed. **c, d.** The same experiments were performed using GalT-mCherry instead of RFP-Rab9. In (**d**), the proportion of GalT-mCherry puncta that colocalize with DAPGreen was analyzed. **e, f.** PentaKO cells were transfected with a plasmid encoding Myc (3×)-tagged- OPTN for 24 hr. Then, these cells were treated with etoposide (10 μM) for 12 hr, and GOMED was analyzed using DAPGreen, and LysoTracker Red DND-99. In (**f**), the number of LysoTracker positive puncta that colocalize with DAPGreen was analyzed. In (**a, c, e**), representative images are shown. White dotted lines indicate the cell shapes. Scale bars = 10 μm. In (**b, d, f**), data are shown as the mean ± SE (n = 10). Comparisons were performed using one-way ANOVA followed by the Tukey's *post-hoc* test. ** $p < 0.001$; ns: not significant. Data are representative of two independent experiments in (**a, c and e**).

Supplementary Figure 5

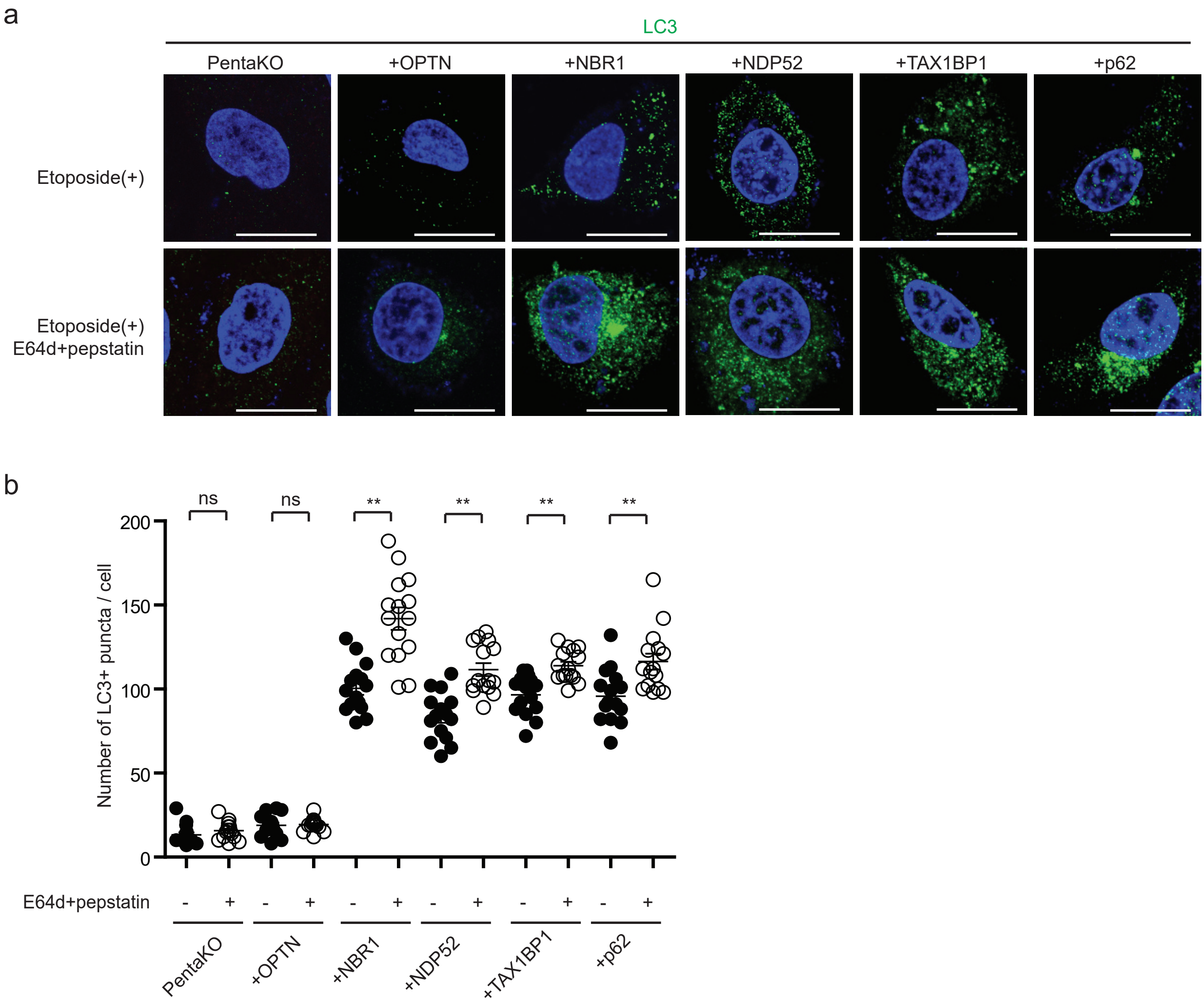

Supplementary Figure 5. Etoposide-induced autophagy is not induced by OPTN expression in PentaKO cells

PentaKO cells were transfected with plasmids encoding Myc (3×)-tagged-adaptor proteins (p62/SQSTM1, NDP52, NBR1, TAX1BP1, or OPTN). After 24 hr, cells were treated with etoposide (10 μM) together with or without E64d (10 μg/mL) / pepstatin (10 μg/mL) for 12 hr, and autophagy was analyzed using LC3 staining (green). Representative images are shown in (**a**). Scale bars = 10 μm. (**b**) Quantitative analysis of LC3 puncta. Data are shown as the mean ± SE (n = 15). Comparisons were performed using one-way ANOVA followed by the Tukey's *post-hoc* test. $^{**}p < 0.01$; ns: not significant. Data are representative of two independent experiments in (**a**).

Supplementary Figure 6

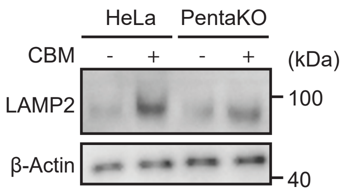

Supplementary Figure 6. CBM increases LAMP2 expression

HeLa cells and PentaKO cells were treated with CBM (1 mM) for 12 hr, were lysed, and immunoblotting was performed for LAMP2. LAMP2 expression was increased upon CBM treatment. Data are representative of two independent experiments.

Supplementary Figure 7

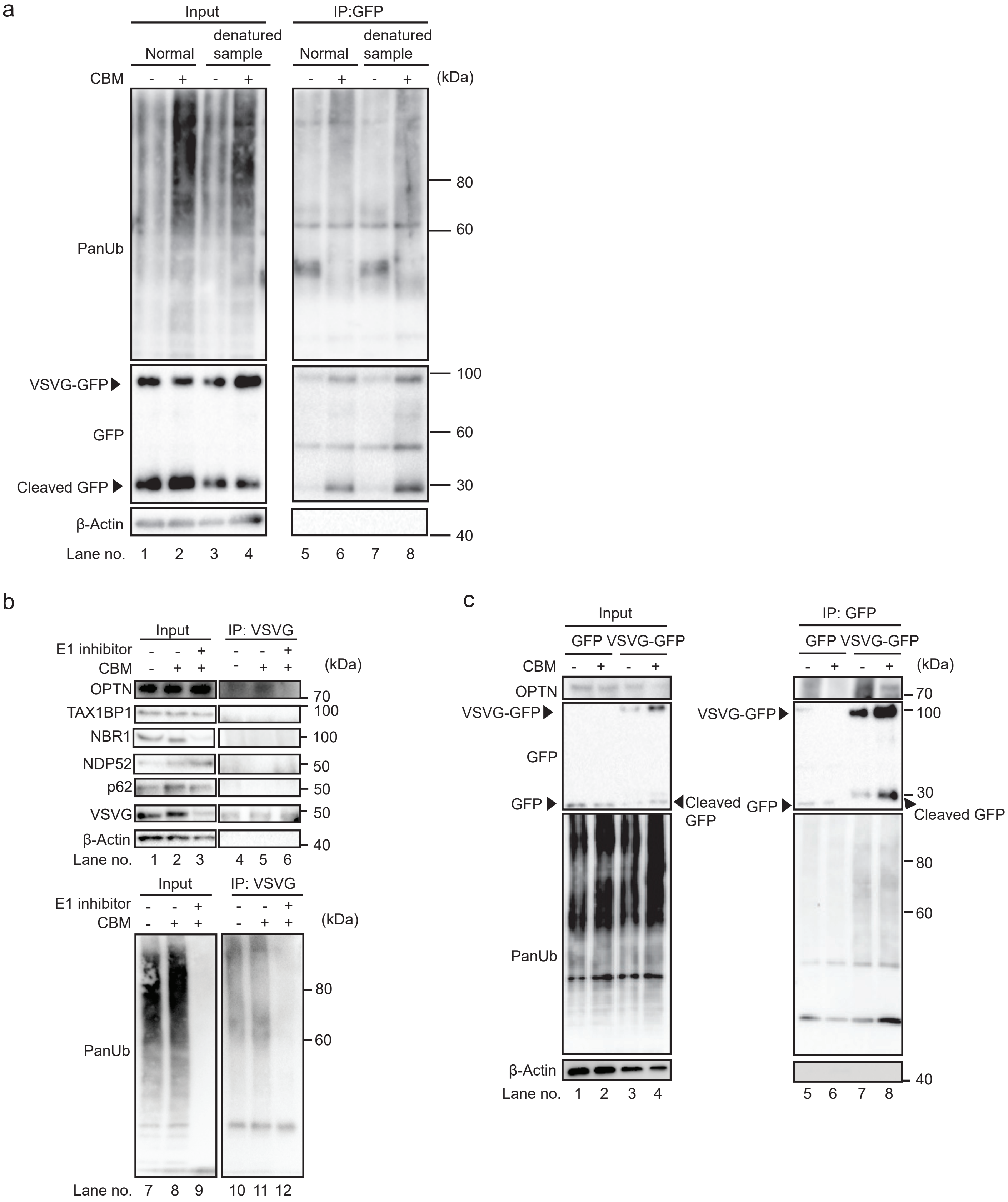

Supplementary Figure 7. CBM induces polyubiquitination of VSVG

a. VSVG-GFP-expressing *Atg5*^{KO} MEFs were treated with CBM (1 mM) for 12hr. Cells were lysed using two different methods; *i.e.*, the regular method and denatured method to avoid nonspecific ubiquitination. The samples were then immunoprecipitated with an anti-GFP antibody. Expression of polyUb, VSVG-GFP, and β-actin in total lysates (Input) and immunoprecipitants (IP: GFP) were analyzed by western blotting. **b.** VSVG was transiently expressed in HEK293T cells. After 24 hr, cells were treated with CBM (1 mM) for 12 hr, or left untreated. Cells were also treated with or without the E1 inhibitor TAK-243 (100 nM) for 1 hr before cell collection. Cells were then immunoprecipitated with an anti-VSVG antibody. The expression of VSVG and adaptor proteins in total lysates (Input) and immunoprecipitants (IP: VSVG) were analyzed by western blotting. **c.** GFP or VSVG-GFP was transiently expressed in HEK293T cells. After 24 hr, cells were treated with CBM (1 mM) for 12 hr, or left untreated. These cells were then lysed and immunoprecipitated with an anti-GFP antibody. The expression of GFP or VSVG-GFP and endogenous OPTN in total lysates (Input) and immunoprecipitants (IP: GFP) were analyzed by western blotting. Data are representative of two independent experiments in (**a, b and c**).

Supplementary Figure 8

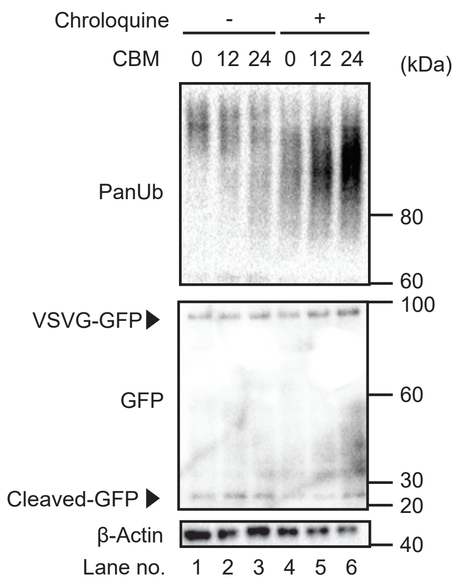

Supplementary Figure 8. Inhibition of VSVG-GFP degradation under CBM stimulation by chloroquine

VSVG-GFP-expressing *Atg5*^{KO} MEFs were treated with CBM (1 mM) in the presence or absence of chloroquine (100 μM). At the indicated times, cells were lysed, and the expression of each protein was analyzed by western blotting. β-actin was used as a loading control. Data are representative of two independent experiments.

Supplementary Figure 9

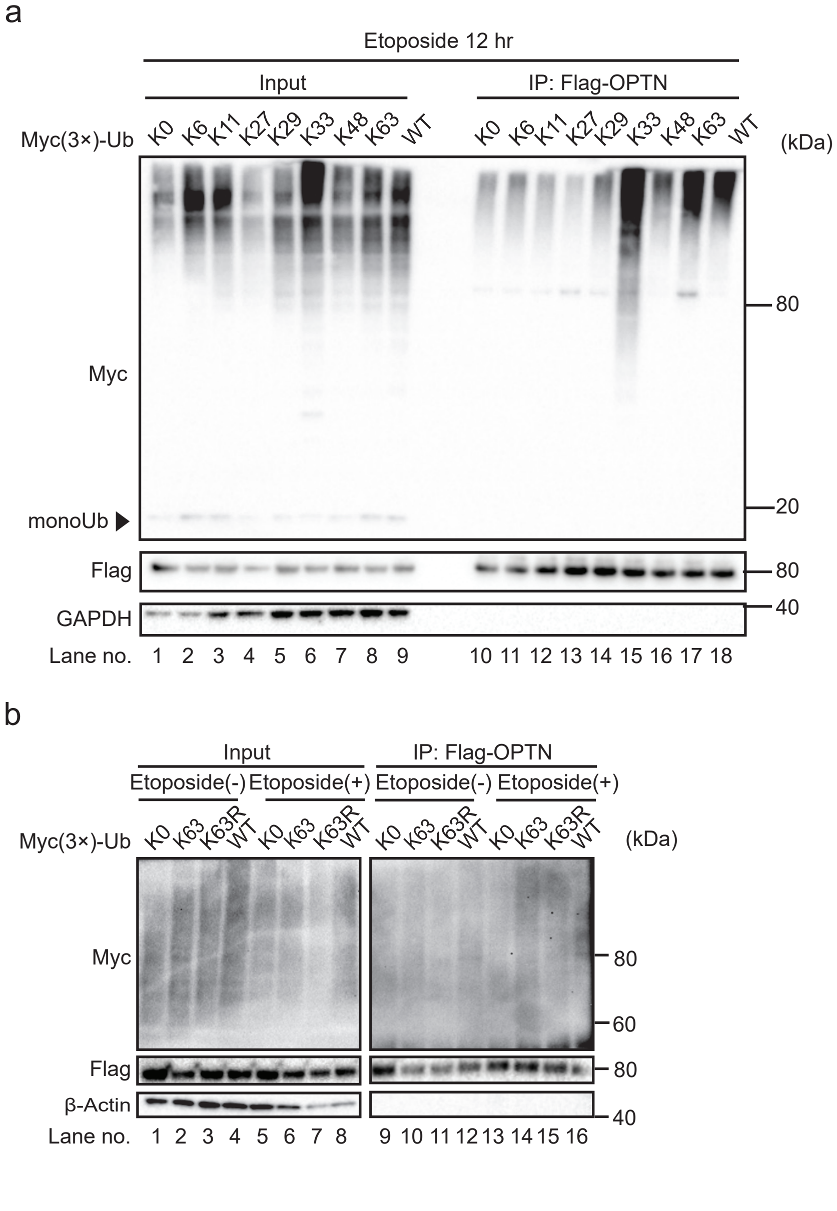

Supplementary Figure 9. Interaction of K33-Ub with OPTN upon etoposide treatment

a. HEK293T cells were transfected with plasmids encoding Flag-OPTN and the indicated Myc (3×)-tagged ubiquitin mutants. After 24 hr, cells were treated with etoposide (10 μM) for 12 hr, and immunoprecipitated with an anti-Flag antibody. Expression of Myc (3×)-tagged Ub mutants, Flag-OPTN, and GAPDH in total lysates (Input) and immunoprecipitants (IP: Flag) were analyzed by western blotting. Note that K33-Ub was mainly coimmunoprecipitated with Flag-OPTN. **b.** HEK293T cells were transfected with plasmids encoding Flag-OPTN and the indicated Myc (3×)-tagged ubiquitin mutants. After 24 hr, cells were treated with etoposide (10 μM) for 12 hr or left untreated. Then, cells were immunoprecipitated with an anti-Flag antibody. Expression of Myc (3×)-tagged Ub mutants, Flag-OPTN, and β-actin in total lysates (Input) and immunoprecipitants (IP: Flag) were analyzed by western blotting. **c.** Similar experiments to **b** were performed using CBM (1 mM) instead of etoposide. Note that K63-polyUb was coimmunoprecipitated with Flag-OPTN upon etoposide treatment (**b**), but not CBM treatment (**c**). Data are representative of two independent experiments in (**a, b and c**).

Supplementary Figure 10

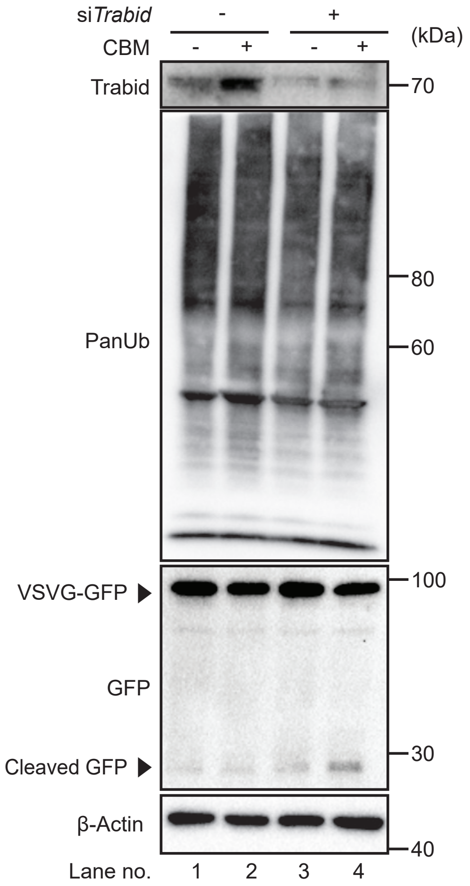

Supplementary Figure 10. Acceleration of GOMED-mediated VSVG-GFP degradation by *Trabid* silencing

VSVG-GFP-expressing *Atg5*^{KO} MEFs were transfected with siRNA against *Trabid*. After 24 hr, cells were treated with CBM (1 mM) for 12 hr, or left untreated. Then, cells were lysed, and subjected to western blotting to analyze the indicated proteins. β-actin was used as a loading control. Data are representative of two independent experiments.

Supplementary Figure 11

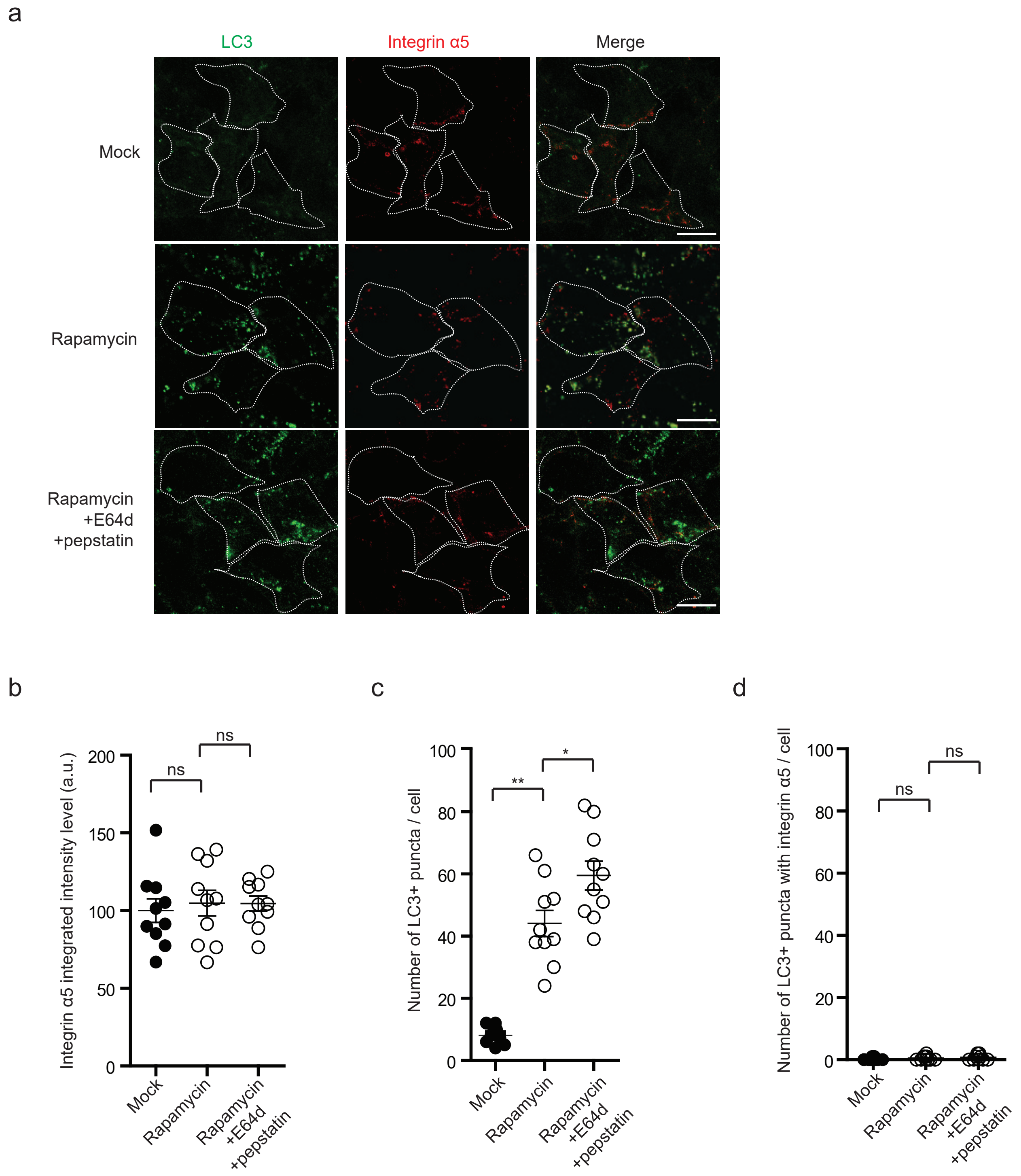

Supplementary Figure 11. Effect of rapamycin on integrin α5 degradation

WT MEFs were treated with rapamycin (1 µM) with or without E64d (10 µg/mL) / pepstatin (10 µg/mL) for 12 hr. Cells were immunostained with anti-LC3 (green) and anti-integrin α5 (red) antibodies. In (**a**), representative images are shown. Bars = 10 µm. The integrated intensity level of integrin α5 (**b**), LC3 puncta (**c**), and the number of LC3 puncta colocalized with integrin α5 (**d**) were analyzed using ImageJ. Data are shown as the mean ± SE (n = 10 cells). Comparisons were performed using one-way ANOVA followed by the Tukey's *post-hoc* test. ns: not significant. Data are representative of two independent experiments.

Supplementary Figure 12

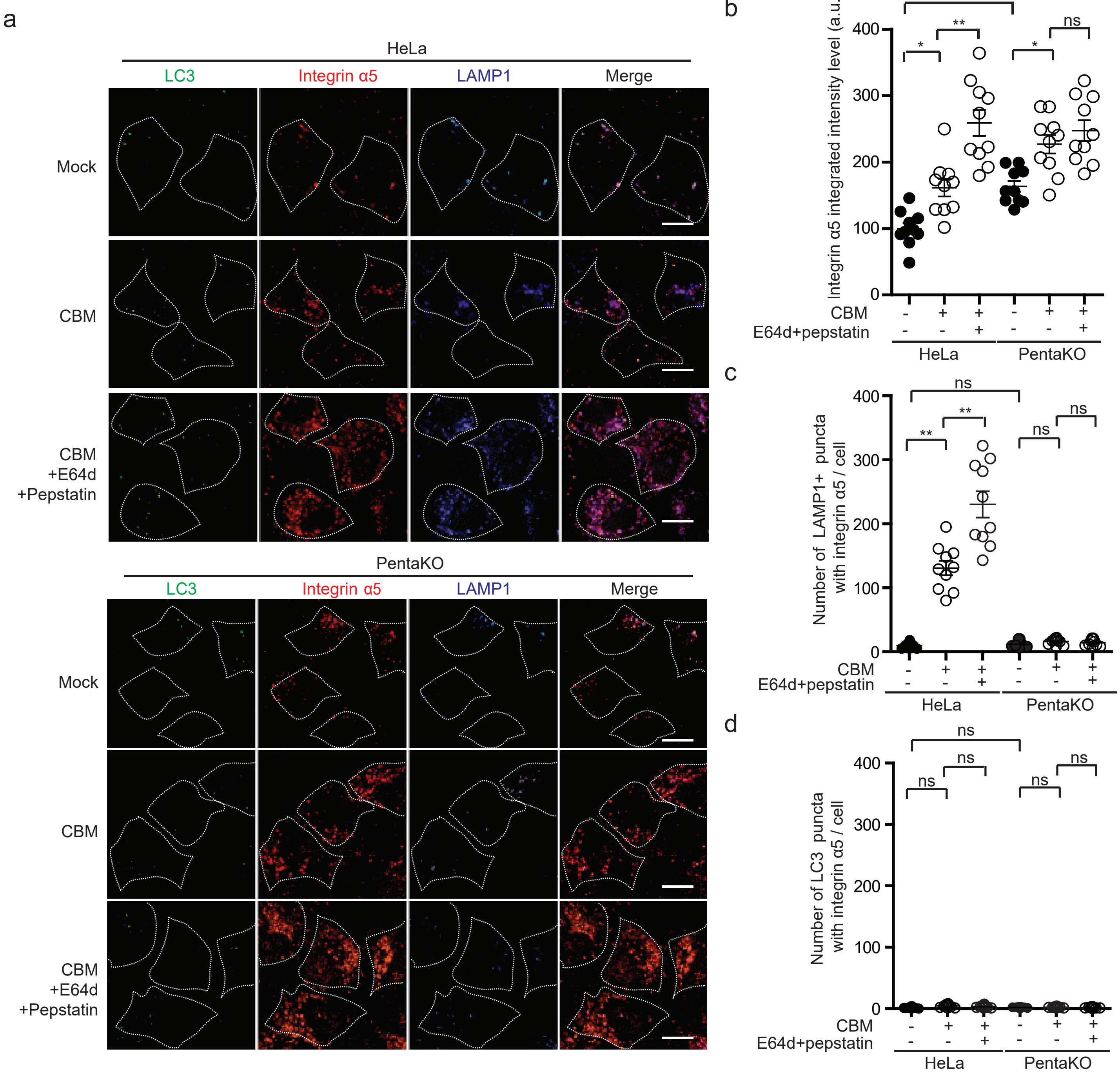

Supplementary Figure 12. Effect of CBM on integrin α5 degradation

HeLa cells and PentaKO cells were treated with CBM (1 mM) with or without E64d (10 μg/mL) / pepstatin (10 μg/mL) for 12 hr. Cells were immunostained with anti-LC3 (green), anti-integrin α5 (red), and anti-LAMP1 (blue) antibodies. In (**a**), representative images are shown. Bars = 10 μm. The integrated intensity level of integrin α5 (**b**), and the number of integrin α5 puncta colocalized with LAMP1 (**c**) and with LC3 (**d**) among total integrin α5 puncta were analyzed using ImageJ. Data are shown as the mean ± SE (n = 10 cells). Comparisons were performed using one-way ANOVA followed by the Tukey's *post-hoc* test. *$p < 0.05$, **$p < 0.01$; ns: not significant. Data are representative of two independent experiments in (**a**).

Supplementary Figure 13

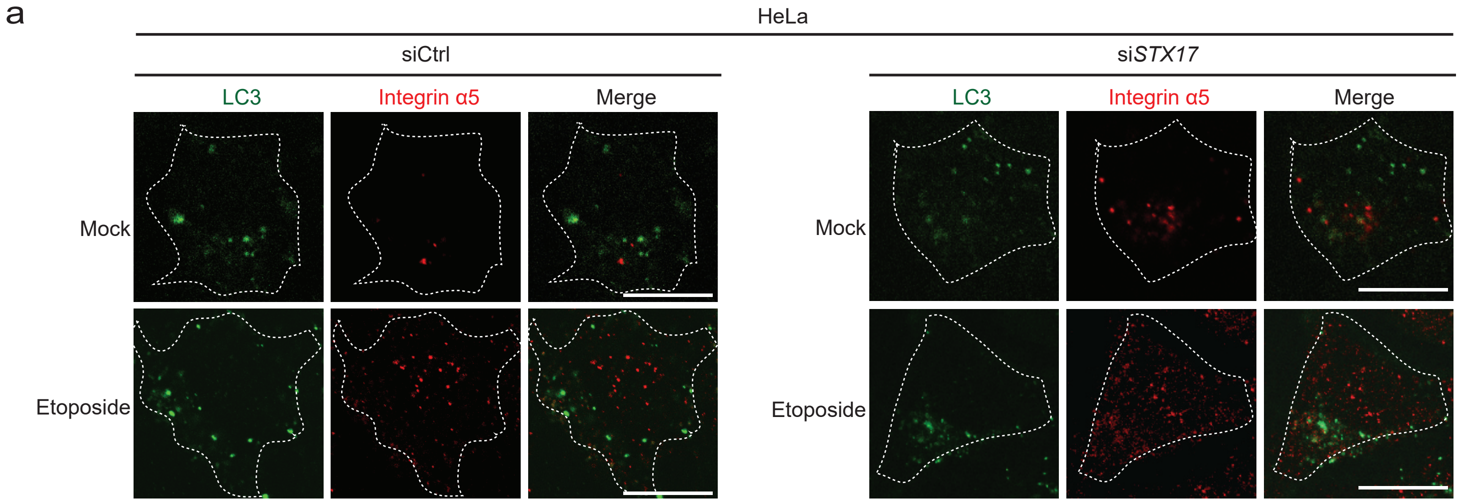

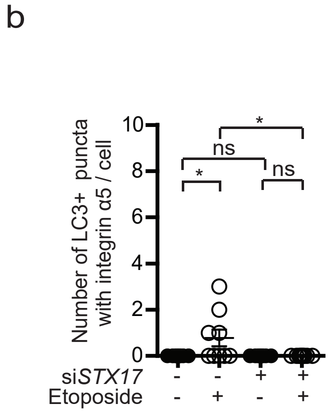

Supplementary Figure 13. Silencing of *STX17* blocks the generation of LC3-integrin α5 double-positive puncta

HeLa cells were added with *STX17* siRNA or control siRNA for 24 hr, and treated with etoposide (10 µM) for 12 hr. Cells were then immunostained with anti-LC3 (green) and anti-integrin α5 (red) antibodies. In **a**, representative images are shown. White dotted lines indicate the cell shapes. Bars = 10 µm. In **b**, the number of integrin α5 puncta colocalizong with LC3 among total integrin α5 puncta was calculated using ImageJ software. Data are shown as the mean ± SE (n = 10 cells). Note that several z-stack images were taken, and quantification was performed on a per-cell basis. Data are representative of two independent experiments in (**a**).

Supplementary Figure 14

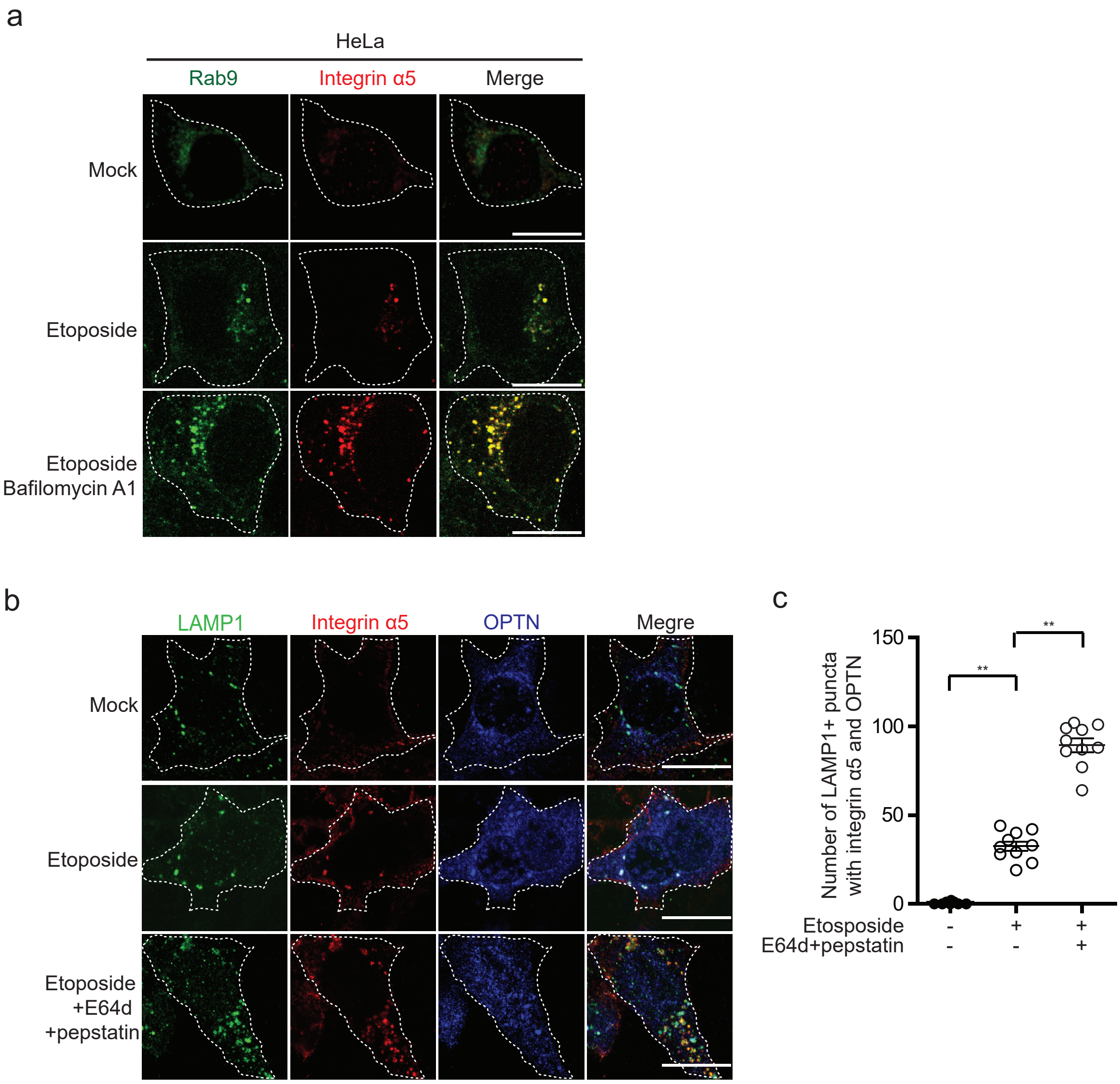

Supplementary Figure 14. Colocalization of integrin α5 with Rab9, Lamp1 and OPTN upon etoposide treatment

a. HeLa cells were treated with etoposide (10 μM) with or without bafilomycin A1 (10nM) for 12 hr. Cells were then immunostained with anti-Rab9 (green) and anti-integrin α5 (red) antibodies White dotted lines indicate the cell shapes. Bars = 10 μm. **b, c.** HeLa cells were treated with etoposide (10 μM) with or without E64d (10 μg/mL) / pepstatin (10 μg/mL) for 12 hr. Cells were then immunostained with anti-LAMP1 (green), anti-integrin α5 (red), and anti-OPTN (blue) antibodies. White dotted lines indicate the cell shapes. Bars = 10 μm. In (**c**), the number of LAMP1 puncta colocalized with integrin α5 and OPTN per cell was calculated. Data are shown as the mean ± SE (n = 10 cells). Note that several z-stack images were taken, and quantification was performed on a per-cell basis. Comparisons were performed using one-way ANOVA followed by the Tukey's *post-hoc* test. $^{**}p < 0.01$; ns: not significant. Data are representative of two independent experiments in (**a and b**).

Supplementary Figure 15

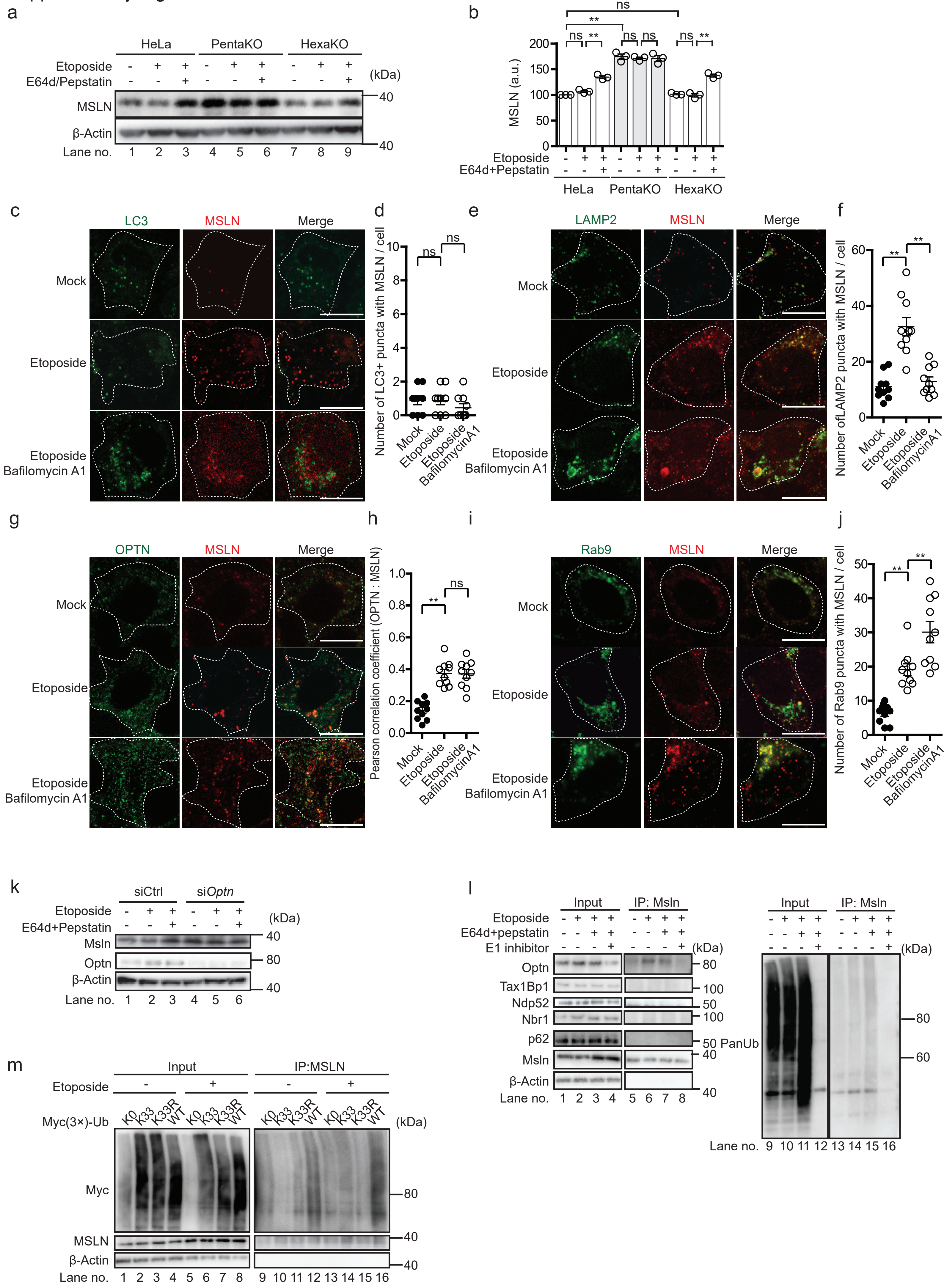

Supplementary Figure 15. Mesothelin (MSLN) is degraded by GOMED via the K33-polyUb-OPTN pathway

a, b. HeLa cells, PentaKO cells, and HexaKO cells were treated with etoposide (10 μM) with or without E64d (10 μg/mL) / pepstatin (10 μg/mL) for 12 hr. Cells were then lysed, and the expression of each protein was analyzed by western blotting. β-actin was used as a loading control. **b.** Semiquantitative analysis of protein expression in the experiment in **a**. Data are shown as the mean ± SE (n = 3). **c–j.** HeLa cells were treated with etoposide (10 μM) with or without bafilomycin A1 (10 nM) for 12 hr. Cells were then immunostained with anti-MSLN (red), and anti-LC3 (**c**), anti-LAMP2 (**e**), anti-OPTN (**g**), or anti-Rab9 (**i**) antibodies. Representative images are shown. White dotted lines indicate the cell shapes. Bars = 10 μm. The number of MSLN puncta colocalizing with LC2 (**d**) , LAMP2 (**f**) and with Rab9 (**j**) were analyzed using ImageJ software. Colocalization of the fluorescence signals of the two molecules (OPTN and MSLN) was also evaluated using Pearson correlation coefficient (**h**). Data are shown as the mean ± SE (n = 10 cells). Note that several z-stack images were taken, and quantification was performed on a per-cell basis. **k.** Analysis of the requirement of Optn for Msln degradation. *Optn* was knocked down by siRNA in*Atg5*^{KO} MEFs. After 24 hr, the expression of each protein was analyzed by western blotting. β-actin was used as a loading control. **l.**Analysis of the interaction of ubiquitinated Msln with Optn. Similar experiments to Fig. 6i were performed using endogenous Msln, instead of endogenous integrin α5. Consistent with integrin α5, Optn was coimmunoprecipitated with Msln upon etoposide treatment. **m.** HEK293T cells were transiently expressed with various Myc (3×)-tagged Ub chains. After 24 hr, cells were treated with etoposide (10 μM) for 12 hr or left untreated, and then immunoprecipitated with an anti-MSLN antibody. Expression of Myc (3×)-tagged Ub chains, MSLN, and β-actin in total lysates (Input) and immunoprecipitants (IP: MSLN) were analyzed by western blotting. Data are representative of two independent experiments in (**a-c, e, g, i and k-m**).

Figure S16

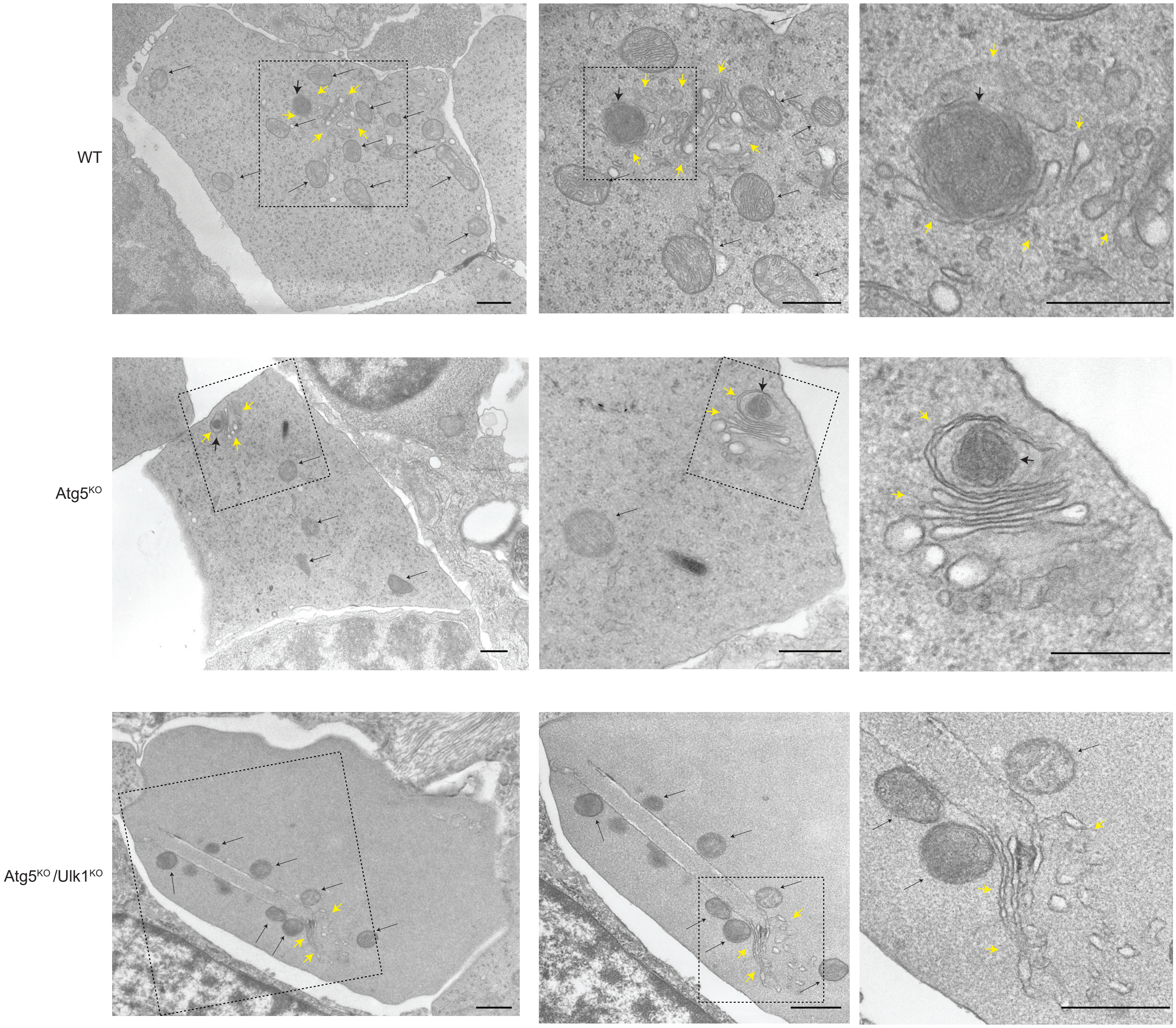

Supplementary Figure 16. Representative EM images of reticulocytes

Representative EM images of reticulocytes obtained from livers of WT, *Atg5*[KO], and *Atg5*[KO]/*Ulk1*[KO] mouse embryos (embryonic day 14.5) are shown in left panels. Magnified images of the squares are shown in the middle panels. Regions of interest (ROIs) are indicated as squares, and their magnified images are shown in the right panels. High-magnification images (right panels) are shown in Fig. 8a. Black and yellow arrows indicate mitochondria and Golgi, respectively. Bars = 0.5 µm.

Supplementary Table 1. Antibody list

name	Campany	Cat. Number	Method	dilution
Rabbit pAb Anti-Optineurin	ABCAM LIMITED	ab23666	WB, IF	1:1000, 1:200
Rabbit mAb Anti-TRAF6BP/TAX1BP1 [EPR13287(B)] - C-terminal	ABCAM LIMITED	ab176572	WB	1:1000
Rabbit pAb Anti-NDP52	GeneTex	GTX115378	WB	1:1000
Rabbit mAb Anti-NBR1 (D2E6)	Cell signaling Technologies	#9891	WB	1:1000
Rabbit pAb Anti-p62 (SQSTM1)	MBL	PM045	WB	1:1000
Mouse mAb Anti-β-Actin (AC-15)	Sigma-Aldrich	A5441	WB	1:3000
Rabbit mAb Anti-Golgin97 (D8P2K)	Cell signaling Technologies	13192	IF	1:200
Rabbit pAb Anti-LAMP1	ABCAM LIMITED	ab24170	IF	1:1000
Rabbit pAb Anti-Integrin α5	Cell signaling Technologies	4705	WB, IF	1:1000, 1:200
Rat mAb Anti-Integrin alpha 5	ABCAM LIMITED	Ab25461	IF	1:200
Rabbit mAb Anti-LC3 (4E12)	MBL	M152-3	IF	1:200
Mouse mAb Anti-LAMP2 (H4B4)	Santa Cruz	sc-18822	IF	1:200
Rat mAb anti-LAMP2 [GL2A7]	ABCAM LIMITED	ab13524	IF	1:200
Anti-GFP(Green Fluorescent Protein) mAb-Magnetic Beads	MBL	D153-11	IP	
Anti-DDDDK-tag mAb-Magnetic Beads	MBL	M185-11R	IP	
Mouse mAb anti- Ubiquitin (P4D1)	Santa cruz	sc-8017	WB	1:200
Rabbit mAb Anti-GFP(D5.1) XP	Cell signaling Technologies	#2956	WB	1:1000
Rabbit pAb Anti-VSV-G	Cell signaling Technologies	81454S	WB, IF	1:1000, 1:100
Mouse mAb anti-GAPDH (6C5)	ABCAM LIMITED	ab8245	WB	1:1000
Mouse mAb Anti-Myc(9E10)	Santa Cruz	SC-40	WB	1:200
Rabbit mAb Anti-FLAG M2	Sigma-Aldrich	F7425	WB	1:1000
Mouse mAb Anti-Tom20(F-10)	Santa Cruz	sc-17764	IF	1:100
Anti Ter119 Beads	BD Biosciences	558536	Isolation	
Mouse mAb Purified Mouse Anti-GM130	BD Biosciences	610822	IF	1:200
Mouse mAb Purified Mouse Anti-GS28	BD Biosciences	611184	IF	1:200
Anti-phospho-Ulk^{Ser746}	Torii et al.[7]	N/A	IF	1:400
Rabbit pAb anti-TRABID(C-13)	Santa Cruz	sc-135536	WB	1:200
Anti-Mesothelin (MSLN) (AA 1-62) antibody, Polyclonal	antibodies-online GmbH	ABIN7371316	WB	1:500
Anti-Mesothelin (MSLN) (AA 400-615) antibody, Polyclonal	antibodies-online GmbH	ABIN7436507	WB, IF	1:500, 1:200
Donkey anti-Rabbit IgG (H+L) Highly Cross-Adsorbed Secondary Antibody, Alexa Fluor™ 488	Thermo Fisher	A-21206	IF	1:200
Donkey anti-Rabbit IgG (H+L) Highly Cross-Adsorbed Secondary Antibody, Alexa Fluor 594	Thermo Fisher	A-21207	IF	1:200
Rabbit anti-Goat IgG (H+L) Cross-Adsorbed Secondary Antibody, Alexa Fluor™ 647	Thermo Fisher	A-21446	IF	1:200
Goat anti-Mouse IgG (H+L) Cross-Adsorbed Secondary Antibody, Alexa Fluor™ 488	Thermo Fisher	A1101	IF	1:200
Donkey anti-Mouse IgG (H+L) ReadyProbes ™ Secondary Antibody, Alexa Fluor™ 594	Thermo Fisher	A21203	IF	1:200
Dynabeads™ Protein G for Immunoprecipitation	Invitrogen	10003D	IP	

Supplementary Table 2. Plasmid list

Recombinant DNA		
Name	Campany	Cat. Number
pCMV-3Tag-2C	Agilent	#240196
GalT-mCherry	Sakurai et al.[39]	N/A
pCMV-3Tag-2C-p62/SQSTM1	This paper	N/A
pCMV-3Tag-2C -NDP52	This paper	N/A
pCMV-3Tag-2C -NBR1	This paper	N/A
pCMV-3Tag-2C -TAX1BP1	This paper	N/A
pCMV-3Tag-2C -OPTN	This paper	N/A
pCMV-3Tag-2C -K0Ub	Nibe et al.[54]	N/A
pCMV-3Tag-2C -K6Ub	This paper	N/A
pCMV-3Tag-2C -K11Ub	This paper	N/A
pCMV-3Tag-2C -K27Ub	This paper	N/A
pCMV-3Tag-2C -K29Ub	This paper	N/A
pCMV-3Tag-2C -K33Ub	Nibe et al. [54]	N/A
pCMV-3Tag-2C -K48Ub	This paper	N/A
pCMV-3Tag-2C -K63Ub	This paper	N/A
pCMV-3Tag-2C -WTUb	This paper	N/A
pCMV-3Tag-2C -K33RUb	Nibe et al. [54]	N/A
Flag-OPTN	Maruyama et al. [65]	N/A
Flag-OPTNQ398X	Maruyama et al. [65]	N/A
Flag-OPTNΔZF	This paper	N/A
Flag-OPTNΔUBA	This paper	N/A
Flag-Optn R1	This paper	N/A
Flag-Optn R2	This paper	N/A
Flag-Optn R3	This paper	N/A
Flag-Optn R4	This paper	N/A
LC3-TagRFP	Sakurai et al.[39]	N/A
pcDNA-VSVG-GFP	Yamaguchi et al.[6]	N/A
pcDNA-VSVG	This paper	N/A
pcDNA3.1-RFP-Rab9	Yamaguchi et al.[6]	N/A

Supplementary Table 3. Primer list

Name	Sequences
Optn-R1 F	AAGCTAAATAATCAAGCTATGAAGGGAAGGTTC
Optn-R1 R	CCAGGCGGACAGCTCCTCGAACCTTCCCTTCAT
Optn-R2 F	ATGTCCCATCAACCTCTGTCTTGTTTAACGGAA
Optn-R2 R	ACAAGGGCTGTCCCCCTTTTCCGTTAAACAAGA
Optn-R3 F	AGACAGTCCCTGATGGAAATGCAATGTAGGCAT
Optn-R3 R	GTCACTGGTTCTTGCCCCATGCCTACATTGCAT
Optn-R4 F	CACCAGCTGAAAGAAGCCATGAAACTGAACAAC
Optn-R4 R	TCGCCCTTTCATAGCTTGGTTGTTCAGTTTCAT
mOPTN-WT-Fwd	GTGGTCCATACTTTCTCCTCAC
mOPTN-WT-Rev	CTATCAGGTCCCTGCTTGTTT
mOPTN-KO-Rev	TAGCTTGGCTGGACGTAAAC
OPTN dUBDF	TGA AAT GAA TGA TGC TTT CGA AGA C
OPTN dUBDR	CGT AGT AAG TAA AGT AGG TAA ACG TC
OPTNiTAAF	CGG CAA TAA CAG CGG AAT ATT CCG ATT CAT TCC
OPTNiTAAR	CCG CTG TTA TTG CCG CCA GTC CCT GTC CTC AGC
K6 onlyF	TTT GTG AAG ACC CTC ACT GGC AAA ACC
K6 only R	GAG GGT CTT CAC AAA GAT CTG CAT GGA
K27 only F	AAT GTC AAA GCC AAA ATT CAA GAC AAG
K27 only R	TTT GGC TCT GAC ATT CTC AAT GGT GTC
K29 only F	AAA GCC AAA ATT CAA GAC AAG GAG GGT
K29 only R	TTG AAT TTT GGC TTT GAC ATT CTC AAT

Supplementary Table 4. Key resources

Name	Campany	Cat. Number
Chemicals, peptides, and recombinant proteins		
Chloroquine	Sigma-Aldrich	C6628
Etoposide	Sigma-Aldrich	E1383
E64d	PEPTIDE INSTITUTE	4321-v
Pepstatin A	Nacalai Tesuque	26436-52
Hoechst 33342, Trihydrochloride	Invitrogen	H3570
1,3-Cyclohexanebis(methylamine), mixture of isomers (CBM)	Sigma-Aldrich	180467
TAK-243 (MLN7243) E1 inhibitor	Takara	HY-100487
GSK’872	Sigma-Aldrich	530389
Rapamycin	Santa Cruz	sc-3504
HBSS(+) with Ca, Mg, without Phenol Red, liquid	Nacalai Tesuque	09735-75
Digitonin (5%)	Thermo Fisher	BN2006
4%-Paraformaldehyde Phosphate Buffer Solution	Nacalai Tesuque	09154-85
Critical commercial assays		
VECTASHIELD® Mounting Medium with DAPI	Vector Laboratories	H-1200-10
DAPGreen - Autophagy Detection	DOJINDO	D676
DAPRed - Autophagy Detection	DOJINDO	D677
LysoTracker™ Red DND-99	Thermo Fisher	L7528
Lipofectamine™ 2000 Transfection Reagent	Thermo FIsher	11668027
Lipofectamine™ RNAiMAX Transfection Reagent	Thermo FIsher	13778075
4D-Nucleofector® X Unit	LONZA	AAF-1003X
P4 Primary Cell 4D-Nucleofector™ X Kit L	LONZA	V4XP-4024
MitoTracker Green FM	Cell signaling Technologies	#9074
MitoTracker Deep Red FM	Cell signaling Technologies	#8778
Pierce™ Mitochondria Isolation Kit	Thermo Fisher	PI89874
PrimeSTAR® Mutagenesis Basal Kit	Takara	R046A
Experimental models: Cell lines		
HEK293T	N/A	N/A
Control HeLaCells	Padman et al. [34]	N/A
PentaKO HeLa Cells	Padman et al.[34]	N/A
Atg5^{KO} MEF	Nishida et al. [4]	N/A
VSVG-GFP expressing *Atg5*^{KO}MEF	Yamaguchi et al.[6]	N/A
Golgi-mCherry expressing *Atg5*^{KO}MEF	Sakurai et al.[39]	N/A
Experimental models: Organisms/strains		
Ulk1^{KO} Mice	Honda et al.[12]	N/A
Optn^{KO}Mice	Kurashige et al. [53]	N/A
Oligonucleotides		
ON-TARGETplus Mouse Optn siRNA	Dharmacon	L-041705-00-0005
ON-TARGETplus Mouse Zranb1(Trabid) siRNA	Dharmacon	L-059888-01-0005
ON-TARGETplus SMARTpool nontargeting siRNA	Dharmacon	D-001810-0X
ON-TARGETplus Mouse Optn (71648) siRNA - Set of 4	Dharmacon	LQ-041705-00-0002
ON-TARGETplus SMARTpool Human STX17 siRNA	Dharmacon	L-020965-01-0005
Software and algorithms		
GraphPad Prism	GraphPad	https://www.graphpad.com/
