# Supplementary material for: Optineurin is an adaptor protein for ubiquitinated substrates in Golgi membrane-associated degradation
Source: Nat Commun. 2025 Oct 20;16:8966. doi: 10.1038/s41467-025-64400-3 (PMC12537940; doi:10.1038/s41467-025-64400-3)
Supplement: Supplementary file 2 — Reporting Summary [file 41467_2025_64400_MOESM2_ESM.pdf]

nature portfolio

Corresponding author(s): Yoichi Nibe-Shirakihara and Shigeomi Shimizu

Last updated by author(s): Jul 14, 2025

Reporting Summary

Nature Portfolio wishes to improve the reproducibility of the work that we publish. This form provides structure for consistency and transparency in reporting. For further information on Nature Portfolio policies, see our Editorial Policies and the Editorial Policy Checklist.

Statistics

For all statistical analyses, confirm that the following items are present in the figure legend, table legend, main text, or Methods section.

n/a	Confirmed	
☐	☒	The exact sample size (n) for each experimental group/condition, given as a discrete number and unit of measurement
☐	☒	A statement on whether measurements were taken from distinct samples or whether the same sample was measured repeatedly
☐	☒	The statistical test(s) used AND whether they are one- or two-sided<br>*Only common tests should be described solely by name; describe more complex techniques in the Methods section.*
☒	☐	A description of all covariates tested
☒	☐	A description of any assumptions or corrections, such as tests of normality and adjustment for multiple comparisons
☐	☒	A full description of the statistical parameters including central tendency (e.g. means) or other basic estimates (e.g. regression coefficient) AND variation (e.g. standard deviation) or associated estimates of uncertainty (e.g. confidence intervals)
☐	☒	For null hypothesis testing, the test statistic (e.g. F, t, r) with confidence intervals, effect sizes, degrees of freedom and P value noted<br>*Give P values as exact values whenever suitable.*
☒	☐	For Bayesian analysis, information on the choice of priors and Markov chain Monte Carlo settings
☒	☐	For hierarchical and complex designs, identification of the appropriate level for tests and full reporting of outcomes
☒	☐	Estimates of effect sizes (e.g. Cohen's d, Pearson's r), indicating how they were calculated

Our web collection on statistics for biologists contains articles on many of the points above.

Software and code

Policy information about availability of computer code

Data collection	FW31S-DT (Olympus)
Data analysis	FW31S-SW (Olympus), Adobe photoshop CS5.1, Adobe Illustrator CS5.1, ImageJ, Excel, Prism 5 (GraphPad)

For manuscripts utilizing custom algorithms or software that are central to the research but not yet described in published literature, software must be made available to editors and reviewers. We strongly encourage code deposition in a community repository (e.g. GitHub). See the Nature Portfolio guidelines for submitting code & software for further information.

Data

Policy information about availability of data

All manuscripts must include a data availability statement. This statement should provide the following information, where applicable:

- Accession codes, unique identifiers, or web links for publicly available datasets
- A description of any restrictions on data availability
- For clinical datasets or third party data, please ensure that the statement adheres to our policy

The data that support the findings of this study are available from the corresponding author upon request

Research involving human participants, their data, or biological material

Policy information about studies with human participants or human data. See also policy information about sex, gender (identity/presentation), and sexual orientation and race, ethnicity and racism.

Reporting on sex and gender	*Use the terms sex (biological attribute) and gender (shaped by social and cultural circumstances) carefully in order to avoid confusing both terms. Indicate if findings apply to only one sex or gender; describe whether sex and gender were considered in study design; whether sex and/or gender was determined based on self-reporting or assigned and methods used. Provide in the source data disaggregated sex and gender data, where this information has been collected, and if consent has been obtained for sharing of individual-level data; provide overall numbers in this Reporting Summary. Please state if this information has not been collected. Report sex- and gender-based analyses where performed, justify reasons for lack of sex- and gender-based analysis.*
Reporting on race, ethnicity, or other socially relevant groupings	*Please specify the socially constructed or socially relevant categorization variable(s) used in your manuscript and explain why they were used. Please note that such variables should not be used as proxies for other socially constructed/relevant variables (for example, race or ethnicity should not be used as a proxy for socioeconomic status). Provide clear definitions of the relevant terms used, how they were provided (by the participants/respondents, the researchers, or third parties), and the method(s) used to classify people into the different categories (e.g. self-report, census or administrative data, social media data, etc.) Please provide details about how you controlled for confounding variables in your analyses.*
Population characteristics	*Describe the covariate-relevant population characteristics of the human research participants (e.g. age, genotypic information, past and current diagnosis and treatment categories). If you filled out the behavioural & social sciences study design questions and have nothing to add here, write "See above."*
Recruitment	*Describe how participants were recruited. Outline any potential self-selection bias or other biases that may be present and how these are likely to impact results.*
Ethics oversight	*Identify the organization(s) that approved the study protocol.*

Note that full information on the approval of the study protocol must also be provided in the manuscript.

Field-specific reporting

Please select the one below that is the best fit for your research. If you are not sure, read the appropriate sections before making your selection.

☒ Life sciences ☐ Behavioural & social sciences ☐ Ecological, evolutionary & environmental sciences

For a reference copy of the document with all sections, see nature.com/documents/nr-reporting-summary-flat.pdf

Life sciences study design

All studies must disclose on these points even when the disclosure is negative.

Sample size	We did not perform statistical analyses to predetermine sample sizes. Sample sizes were based on empirical data from pilot experiments and similar published our studies (Torii et al., 2020, doi.org/10.1038/s41467-020-15577-2, Yamaguchi et al., 2016, doi: 10.15252/embj.201593191, Honda et al., 2014,doi.org/10.1038/ncomms5004). Sample sizes are described in Methods and figure legends.
Data exclusions	No data was excluded from analysis.
Replication	Western blot analyses were conducted at least in duplicate. For each representative image, each experiment was repeated at least three times under similar condition. The number of replicates for each experiments is clearly presented in the figure legends.
Randomization	A batch of identical cultured cells were randomly allocated to treatment groups and control groups. Animals were randomly put into cages and randomly allocated to experimental groups.
Blinding	Blinding was not possible because the experiments were performed by a single researcher.

Reporting for specific materials, systems and methods

We require information from authors about some types of materials, experimental systems and methods used in many studies. Here, indicate whether each material, system or method listed is relevant to your study. If you are not sure if a list item applies to your research, read the appropriate section before selecting a response.

Materials & experimental systems

n/a	Involved in the study
	[x] Antibodies
	[x] Eukaryotic cell lines
[x]	Palaeontology and archaeology
	[x] Animals and other organisms
[x]	Clinical data
[x]	Dual use research of concern
[x]	Plants

Methods

n/a	Involved in the study
[x]	ChIP-seq
[x]	Flow cytometry
[x]	MRI-based neuroimaging

Antibodies

Antibodies used

The antibodies used are listed in Materials.
Here we state in order: Antibody name; clone (Company; Catalogue number; Dilution)
Rabbit pAb Anti-Optineurin (ABCAM LIMITED, ab23666, 1:1000 for immunoblotting, 1:200 for immunostaining)
Rabbit mAb Anti-TRAF6BP/TAX1BP1 [EPR13287(B)] - C-terminal(ABCAM LIMITED, ab176572, 1:1000 for immunoblotting)
Rabbit pAb Anti-NDP52 (GeneTex , GTX115378, 1:1000 for immunoblotting)
Rabbit mAb Anti-NBR1 (D2E6)(Cell signaling Technologies, #9891, 1:1000 for immunoblotting)
Rabbit pAb Anti-p62 (SQSTM1) (MBL, PM045, 1:1000 for immunoblotting)
Mouse mAb Anti-β-Actin (AC-15)(Sigma-Aldrich, A5441, 1:3000 for immunoblotting)
Rabbit mAb Anti-Golgin97 (D8P2K) (Cell signaling Technologies, 13192, 1:200 for immunostaining)
Rabbit pAb Anti-LAMP1(ABCAM LIMITED, ab24170, 1:1000 for immunostaining)
Rabbit pAb Anti-Integrin α5(Cell signaling Technologies, 4705, 1:1000 for immunoblotting, 1:200 for immunostaining)
Rat mAb Anti-Integrin alpha 5[5H10-27] (Phycoerythrin)(ABCAM LIMITED, Ab25461, 1:200 for immunostaining)
Rabbit mAb Anti-LC3 (4E12)(MBL, M152-3, 1:200 for immunostaining)
Mouse mAb Anti-LAMP2 (H4B4)(Santa Cruz, sc-18822 , 1:200 for immunoblotting, 1:100 for immunostaining)
Rat mAb anti-LAMP2 [GL2A7](ABCAM LIMITED, ab13524, 1:200 for immunostaining)
Mouse mAb anti- Ubiquitin (P4D1) (Santa cruz, sc-8017, 1:200 for immunoblotting)
Rabbit mAb Anti-GFP(D5.1) XP (Cell signaling Technologies, #2956, 1:1000 for immunoblotting)
Rabbit pAb Anti-VSV-G(Cell signaling Technologies, 81454S, 1:1000 for immunoblotting, 1:100 for immunoprecipitation)
Mouse mAb anti-GAPDH (6C5)(ABCAM LIMITED, ab8245, 1:1000 for immunoblotting)
Mouse mAb Anti-Myc(9E10) (Santa Cruz, SC-40, 1:100 for immunostaining, 1:200 for immunoblotting)
Rabbit mAb Anti-FLAG M2(Sigma-Aldrich, F7425, 1:1000 for immunoblotting)
Mouse mAb Anti-Tom20(F-10) (Santa Cruz, sc-17764, 1:100 for immunostaining)
Mouse mAb Purified Mouse Anti-GM130(BD Biosciences Pharmingen, 610822, 1:200 for immunostaining)
Mouse mAb Purified Mouse Anti-GS28(BD Biosciences Pharmingen, 611184, 1:200 for immunostaining)
Anti-phospho-UlkSer746(self-produced previously (Torii et al., 2020, doi.org/10.1038/s41467-020-15577-2), (Eurofins, rabbit, 1:400 for immunostaining)
Rabbit pAb anti-TRABID(C-13)(Santa Cruz, sc-135536, 1:200 for immunoblotting)
Anti-Mesothelin (MSLN) (AA 1-62) antibody, Polyclona, antibodies-online GmbH, ABIN7371316, 1:500 for immunoblotting)
Anti-Mesothelin (MSLN) (AA 400-615) antibody, Polyclonal, antibodies-online GmbH, ABIN7436507,1:500 for immunoblotting, 1:200 for immunostaining, 1:100 for immunoprecipitation)

Validation

Validation statement of each primary antibody can be found on the manufacturer's website.
The antibodies that have been validated by the suppliers for specific purpose (for example, mouse samples using western blot and/or immunofluorescence) were purchased for our experiments.
Anti-phospho-UlkSer746(self-produced previously (Torii et al., 2020, doi.org/10.1038/s41467-020-15577-2))was validated previously.

Eukaryotic cell lines

Policy information about cell lines and Sex and Gender in Research

Cell line source(s)	MEFs were generated from embryos on embryonic day 14.5 by immortalization with the SV40 T antigen.<br>PentaKO, HexaKO, and HeLa cells (Lazarou et al., Nature, 2015) were kindly provided by Dr. Michael Lazarou.
Authentication	MEFs were analyzed by PCR using specific primers to detect genome-editing.
Mycoplasma contamination	We confirm that all cell lines tested were negative for mycoplasma contamination. All cell lines are checked by staining with Hoechst and obsevation. In addition, some cells are checked by electron microscopy.
Commonly misidentified lines (See ICLAC register)	No commonly misidentified cell lines were used

Animals and other research organisms

Policy information about studies involving animals; ARRIVE guidelines recommended for reporting animal research, and Sex and Gender in Research

Laboratory animals	57BL/6 mice were purchased from Japan CLEA (Tokyo, Japan). The generation of Ulk1KO mice and OptnKO mice has been described previously(Honda et al., 2014,doi.org/10.1038/ncomms5004, Kurashige et al., 2021, doi: 10.1016/j.nbd.2020.105215.). Mice were bred on a 12-h light/12-h dark cycle at approximately 23°C and 40% relative humidity at the Laboratory for Recombinant Animals of Institute of Science Tokyo, Tokyo, Japan. This animal facility is operated according to the NIH guidelines. The Institute of Science Tokyo Ethics Committee for Animal Experiments approved all experiments in this study, and all experiments were performed according to their regulations.
Wild animals	The study did not involve wild animals.
Reporting on sex	In this study, sex differences in mice were not taken into consideration. Additionally, since P1 mice were used, it was visually challenging to distinguish between males and females.
Field-collected samples	The study did not involve samples collected from the field.
Ethics oversight	The Insititute of Science Tokyo Ethics Committee for Animal Experiments approved all experiments in this study.

Note that full information on the approval of the study protocol must also be provided in the manuscript.

Plants

Seed stocks	*Report on the source of all seed stocks or other plant material used. If applicable, state the seed stock centre and catalogue number. If plant specimens were collected from the field, describe the collection location, date and sampling procedures.*
Novel plant genotypes	*Describe the methods by which all novel plant genotypes were produced. This includes those generated by transgenic approaches, gene editing, chemical/radiation-based mutagenesis and hybridization. For transgenic lines, describe the transformation method, the number of independent lines analyzed and the generation upon which experiments were performed. For gene-edited lines, describe the editor used, the endogenous sequence targeted for editing, the targeting guide RNA sequence (if applicable) and how the editor was applied.*
Authentication	*Describe any authentication procedures for each seed stock used or novel genotype generated. Describe any experiments used to assess the effect of a mutation and, where applicable, how potential secondary effects (e.g. second site T-DNA insertions, mosiacism, off-target gene editing) were examined.*
